# Update of transmission modelling and projections of *gambiense* human African trypanosomiasis in the Mandoul focus, Chad

**DOI:** 10.1186/s40249-022-00934-8

**Published:** 2022-01-24

**Authors:** Kat S. Rock, Ching-I Huang, Ronald E. Crump, Paul R. Bessell, Paul E. Brown, Inaki Tirados, Philippe Solano, Marina Antillon, Albert Picado, Severin Mbainda, Justin Darnas, Emily H. Crowley, Steve J. Torr, Mallaye Peka

**Affiliations:** 1 Mathematics Institute, University of Warwick, Academic Loop Road, Coventry, CV4 7AL UK; 2 Zeeman Institute for Systems Biology & Infectious Disease Epidemiology Research (SBIDER), University of Warwick, Academic Loop Road, Coventry, CV4 7AL UK; 3 Independent Consultant, Edinburgh, UK; 4 Department of Vector Biology, Liverpool School of Tropical Medicine, Liverpool, UK; 5 Institut de Recherche pour le Développement, UMR INTERTRYP IRD-CIRAD, Université de Montpellier, 34398 Montpellier, France; 6 Swiss Tropical and Public Health Institute, Basel, Switzerland; 7 University of Basel, Basel, Switzerland; 8 Foundation for Innovative New Diagnostics (FIND), Geneva, Switzerland; 9 Programme National de Lutte contre la Trypanosomiase Humaine Africaine (PNLTHA), Moundou, Chad

**Keywords:** *Gambiense* human African trypanosomiasis (gHAT), Modelling, Elimination of transmission, Validation, Tsetse, Vector control, *Glossina*, Diagnostics

## Abstract

**Background:**

In recent years, a programme of vector control, screening and treatment of *gambiense* human African trypanosomiasis (gHAT) infections led to a rapid decline in cases in the Mandoul focus of Chad. To represent the biology of transmission between humans and tsetse, we previously developed a mechanistic transmission model, fitted to data between 2000 and 2013 which suggested that transmission was interrupted by 2015. The present study outlines refinements to the model to: (1) Assess whether elimination of transmission has already been achieved despite low-level case reporting; (2) quantify the role of intensified interventions in transmission reduction; and (3) predict the trajectory of gHAT in Mandoul for the next decade under different strategies.

**Method:**

Our previous gHAT transmission model for Mandoul was updated using human case data (2000–2019) and a series of model refinements. These include how diagnostic specificity is incorporated into the model and improvements to the fitting method (increased variance in observed case reporting and how underreporting and improvements to passive screening are captured). A side-by-side comparison of fitting to case data was performed between the models.

**Results:**

We estimated that passive detection rates have increased due to improvements in diagnostic availability in fixed health facilities since 2015, by 2.1-fold for stage 1 detection, and 1.5-fold for stage 2. We find that whilst the diagnostic algorithm for active screening is estimated to be highly specific (95% credible interval (*CI*) 99.9–100%, Specificity = 99.9%), the high screening and low infection levels mean that some recently reported cases with no parasitological confirmation might be false positives. We also find that the focus-wide tsetse reduction estimated through model fitting (95% *CI* 96.1–99.6%, Reduction = 99.1%) is comparable to the reduction previously measured by the decline in tsetse catches from monitoring traps. In line with previous results, the model suggests that transmission was interrupted in 2015 due to intensified interventions.

**Conclusions:**

We recommend that additional confirmatory testing is performed in Mandoul to ensure the endgame can be carefully monitored. More specific measurement of cases, would better inform when it is safe to stop active screening and vector control, provided there is a strong passive surveillance system in place.

**Graphical Abstract:**

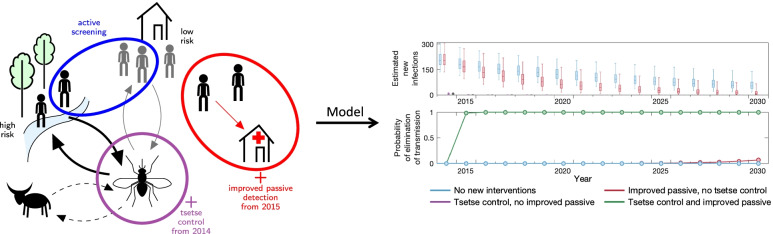

**Supplementary Information:**

The online version contains supplementary material available at 10.1186/s40249-022-00934-8.

## Background

A new World Health Organization (WHO) roadmap [1] has set out control, elimination or eradication goals to be achieved by 2030 for 20 different neglected tropical diseases (NTDs)—a collection of mainly infectious diseases affecting some of the poorest and most marginalised populations globally. Amongst them is *gambiense* human African trypanosomiasis (gHAT), a vector-borne, parasitic infection which has a goal of elimination of transmission (EOT) by 2030 following the success indicated by decline in global disease reporting in the last two decades [2]. Formerly, this disease—caused by *Trypanosoma brucei gambiense* and transmitted by tsetse (*Glossina* spp.) to humans—impacted 36 countries across West and Central African [3], usually resulting in death without detection and treatment of those infected. Now only 24 countries are considered endemic—at least marginal risk—and, of these, six have reported only tens of cases for the last five years and nine have reported single figures [2].

gHAT is a slow progressing disease with early (stage 1) infection causing relatively mild or non-specific symptoms such as headache or fever [4] with the parasite found in blood and lymph fluid. Following a period of around one to two years [5], infection penetrates the blood–brain barrier where it causes late stage (stage 2) disease and the parasite may be found in cerebrospinal fluid (CSF). During stage 2 patients may suffer neurological symptoms including, but not limited to, neuropsychiatric symptoms, sleep disturbance, abnormal gait or movement, and eventually death [4, 6]. gHAT is not vaccine preventable nor is it currently possible to use mass distribution of drugs to treat infection. Patients will only be treated following identification of the parasite in the blood, lymph system or CSF, or in some settings as a result of strong serological evidence. Consequently there is a large diagnostic component to medical interventions and cases must be identified either via mass screening of village populations in endemic areas (active screening) or rely on fixed health care facilities, known as “passive” detection. A critical difference between passive and active screening is that all individuals are screened in active screening irrespective of their symptomatic status whilst passive screening only screens those with gHAT symptoms. Due to the non-severe clinical signs in stage 1 and the differential diagnosis with malaria, the detection rate in passive stage 2 is higher than that of stage 1 as symptoms are more acute and patients seek medical treatment at health facilities [5]. At this point, a patient may have been infected and potentially infectious for several years. More details on the differences of active and passive screening can be found elsewhere [4].

Initial screening for gHAT is performed using either the card agglutination test for trypanosomiasis (CATT) or rapid diagnostic tests (RDTs) followed by various microscopy techniques to establish the presence of active infection. Following diagnosis it had to be established whether the infection was stage 1 or stage 2—determined by either the presence of trypanosomes or more than 5 white blood-cells per $\upmu \mathrm{l}$ in CSF. Up until now, this staging has been necessary for the administration of stage-specific drugs. In recent years the treatments have been intravenously-administered pentamidine for stage 1 and nifurtimox–eflornithine combination treatment (NECT) for stage 2, over the course of 10–14 days. This has changed in recent years with the introduction of fexinidazole which has obviated the requirement for staging in all but young patients and severely infected patients [6].

Detection and treatment of infected people has been the primary method to control disease and reduce transmission by reducing the time people spend infected, and thereby limiting opportunities for transmission back to tsetse vectors. However, the vector-borne nature of transmission provides other complementary options for control, including targeting the flies directly. A range of different ways to reduce tsetse populations exist, including ground and aerial spraying, insecticide-treated cattle, and insecticide-impregnated targets [7, 8]. Although we are mainly preoccupied with gHAT infection causing disease in humans, there remain questions about the potential role of animals in gHAT transmission cycles. Often gHAT has been described as an anthroponotic infection, however evidence demonstrates that gHAT infection can be found in domestic and wild animals in various gHAT foci [9]. Unfortunately the sparsity of data on animal infection and general very low prevalence of infection in humans means that it is challenging to conclude directly from empirical data whether and how much animals contribute to transmission. Despite this, we may still hypothesise that there could be non-human animal–tsetse–human transmission occurring.

The large reductions in cases globally between 1998 and today are broadly attributed to tools used for diagnosis and treatment of infections [2], including CATT and NECT. However, in the last decade the advances in RDTs and vector control have been accelerating progress in specific regions [10–13]. Chad is one of the countries now reporting very small numbers of cases—in 2002 there was a peak of 715 cases of gHAT reported, making it the country with the fifth highest burden, but in 2019 there were just 16 cases, falling to seventh place relative to all endemic countries [14]. Reductions between 2000 and 2013 are attributed to active screening of at-risk populations and passive screening in endemic foci, however in Chad’s main focus of Mandoul, additional interventions began in 2014 and 2015 to accelerate towards zero. Vector control started in 2014 using Tiny Targets—with a measured tsetse density reduction of 99% after 4 months, and in 2015 the passive screening system was fortified by increasing the number of fixed health facilities with RDTs [12].

Across the continent, combinations of screening, treatment and sometimes vector control are being used to bring down remaining disease burden. The primary strategy against gHAT has been the use of screening and treatment; annual active screening of the population is recommended by WHO in villages where cases have been detected within the past three years [4] and this has worked well to greatly reduce infection incidence in many settings [1]. However, vector control tools, which complement screening activities, have been gradually rolled out in key gHAT hotspots over the last decade and are now being deployed in all extant foci of Côte d’Ivoire, Guinea and Uganda [13] with effectiveness ranging from 80 to 97% tsetse reduction [10, 15, 16].

Success and speed of infection reduction are governed by a variety of factors including geographical extent of foci, tsetse habitat, strength of the local health system [17], disruptions in control activities due to political unrest, other disease outbreaks (including Ebola in Guinea [18] and coronavirus disease 2019 (COVID-19) in all settings [19, 20]), or lack of resources [21]. For example, in the Democratic Republic of Congo (DRC) the large geographic spread of endemic gHAT foci coupled with financial and logistical constraints currently limit the capacity to perform country-wide vector control. Some regions additionally have access challenges associate with limited infrastructure or political instability [22]. Therefore, current efforts in vector control for DRC target the regions of highest incidence [20, 23]. Fortunately, the focus of gHAT in Mandoul presents a unique opportunity for gHAT control as it is a relatively small area with a relatively small population. What is more, the tsetse habitat is a single discrete area with no scope for reinvasion or importation of vectors from other areas.

Previous modelling work suggested that it was likely that transmission was already interrupted by 2015 [12], with 62.8% (95% credible interval (*CI*) 59–66%) of the transmission reduction due to vector control. However, in the present study we question whether this is consistent with the low-level but persistent case reporting still occurring in the focus. There are numerous other questions surrounding transmission and reporting in the Mandoul focus: (i) given there were six reported cases in 2018 and 11 in 2019, what does this tell us about underlying transmission? (ii) when can we expect to observe zero cases reported? (iii) can active screening and vector control be stopped without risking recrudescence?

In the present study we critique and refine the previous modelling work to reassess likely transmission, and update predictions until 2030 utilising a further four years of case data. Specifically, we improve upon our previous transmission model by allowing for false positive case reporting, increasing observational variation in simulated case reporting, and using additional years’ data to parameterise improvements in passive detection and effectiveness of vector control. In addition our updated model utilises a revised statistical fitting methodology. Both the original model and update model include the use of an ensemble model approach where we assess statistical support for model variants with and without animal transmission and, based on this support, weight our modelling results accordingly. In order to ensure clear, reproducible and rigorous modelling for this study we use the Policy-Relevant Items for Reporting Models in Epidemiology of Neglected Tropical Diseases (PRIME-NTD) checklist [24]. This completed checklist can be found in our Additional file 1 (section S9) and includes details on stakeholder engagement, model documentation, description of data used, communicating uncertainty and testable model outcomes. New results are also available via an interactive graphical user interface (https://hatmepp.warwick.ac.uk/Mandoulfitandproject/v1/).

## Methods

### Data

To quantify the transmission and case reporting of gHAT in the Mandoul focus of Chad, we utilised the WHO HAT Atlas data from 2000 to 2018 [2]. Records within this data set were annually aggregated by mode of screening/detection (i.e. active and passive), and location. In a single record the number of people screened, cases identified and staging (if known) were recorded. In order to fit our model to the focus-level data we aggregated all locations with the Mandoul focus together, but retained information on the number of active (stage 1, stage 2 and stage unknown) cases, passive (stage 1, stage 2 and stage unknown) cases, and the number of people actively screened in each year. To supplement these data, we also used 2019 case and screening data provided by the national control programme of Chad (Programme National de Lutte contre la Trypanosomiase Humaine Africaine; PNLTHA-Chad). The geographical location and approximate extent of the Mandoul focus and other foci in Chad are presented in Additional file 1: Fig. S1.

In Chad, case identification follows an algorithm involving serological screening using CATT (in vehicle-based active screening (AS)) and RDT (in passive screening (PS) and motorcycle-based AS). Confirmation of the presence of circulating parasites is by various microscopy techniques, supplemented in 2017 by the highly-sensitive mini anion exchange centrifugation technique (mAECT). However, in the absence of positive microscopy, cases are also confirmed by clinical signs and positive results by CATT on diluted serum. For some suspects additional (but not final) diagnostic data is obtained using loop-mediated isothermal amplification (LAMP).

### The gHAT model

Mechanistic transmission modelling is one way to represent dynamic changes in the spread of an infection over time. It can account for variable interventions (in particular AS), or introduction of a new strategy (such as improved PS and vector control (VC)). Once calibrated to data, models can be used to predict what we might expect to happen in the future if the current intervention strategy is continued, or if changes are made.

The Warwick gHAT model has been developed over the past five years to represent the known biology of transmission between humans and tsetse (and possibly non-human animals), and to capture key gHAT intervention strategies used in both the Democratic Republic of Congo and Chad. Since its original development [25] several modifications have been made based on continual fitting to different longitudinal data sets, followed by assessment and refinement [12, 26–28]. In the present study we specifically discuss the evolution of the Warwick gHAT model since it was originally fitted to data from the Mandoul focus [12] and take steps to update the previous fits and provide new projections for the future under different strategies.

### Assessment and update of model fits

In order to quantitatively assess previous model projections and refine them we take three steps.

#### Step 1: Previous model projections corrected for new active screening

First we use the previously developed model code and posterior parameterisation from fitting to data from 2000 to 2013 [12] and use the AS numbers from 2014 to 2019 to update projections for those six years. In Mahamat et al., screening numbers for 2014 and 2015 were known, but numbers for 2016–2019 were not. Large differences between assumed screening levels and actual screening levels can have a substantial impact on model projections—if there is no screening there would be no active cases, whereas there could be a high number of cases if large proportions of the population were screened. In Mahamat et al., it was assumed that the screening numbers for 2016–2019 would be 27,265 each year (the same as in 2015), whereas new data from the WHO HAT Atlas and PNLTHA-Chad are somewhat lower—22,0071, 18,144, 18,083 and 12,640 for each of the years respectively. This first step enables us to compare new data with old model predictions (adjusted for the correct screening numbers). In line with the previous results, we utilised ensemble results from four different model variants, two of which include animal reservoirs (see Additional file 1 for more details on the previous model).

Following fitting in Mahamat et al. [12], counterfactual scenarios (CFSs) were run for 2014–2019 to estimate the impact of intensified interventions (referring to the introduction of VC in 2015 and improvements in PS in the Mandoul focus from 2015). For CFSs considered here, we simulated what would have been expected if (a) neither VC nor improved PS had been introduced in 2014 and 2015, (b) if the only change was improved PS, and (c) if the only change was VC. These three scenarios were compared to the “actual” strategy (with both VC and improved PS) in order to assess the relative impact of the different intervention types in reducing transmission.

#### Step 2: New model fit for 2000–2013

Since the publication of Mahamat et al. [12], there have been several refinements to the underlying gHAT model used. Amongst these, the model is now able to capture greater variance in observed case reporting (overdispersion) which was originally developed for Model W in Castano et al. [26], and the model fitting procedure was improved by using informative priors which take previous biological beliefs about the parameterisation into account (e.g. the high-risk group in the population is likely a similar proportion to the proportion of working age men and the basic reproduction number, $R_0$, is probably only slightly above 1) [28]. Furthermore modelling of the improvement to PS (used from 2015 onwards to simulate increased RDT usage in Mandoul) has a slightly different formulation: from a discrete jump in detection rates to the use of a steep logisitic function, although the consequence of this change is expected to be minimal (see Additional file 1, section S2.3). In the previous model, underreporting in PS was assumed to occur with both stage 1 and 2 infections, however subsequent updates now assume that true stage 1 infection either leads to reported cases or progression to stage 2. Stage 2 infections can still lead to underreported deaths, with the parameter *u* dictating the (passive) reporting probability of stage 2 infections not picked up by AS.

The specificity of the diagnostic algorithm in previous modelling was assumed to be 100%, whereas PNLTHA-Chad currently allow for treatment based on either a serological or parasitological diagnosis. Patients who are only positive by serology must remain positive after a repeat CATT test is performed with 1:8 dilution, rather than only on the initial CATT on whole blood, and these patients are termed “strongly” seropositive cases. The inclusion of these strongly seropositive individuals as cases has the potential to miss fewer remaining infections who might otherwise be false negative in parasitology, and therefore treat these people and shrink the remaining infectious reservoir. However, it also risks over-diagnosis of cases (false positives) due to the high number of people screened per year, even with a high estimated specificity of over 99% for an algorithm including CATT 1:16 case confirmation (95% *CI* 97.7–99.7%, Spec = 99.1% [29], 95% *CI* 99.2–99.5%, Spec = 99.4% [30], and > 99.6% [31]). As infection continues to decline in Mandoul—and globally—the positive predictive value of tests is reduced and eventually false positives may outnumber true positives without parasitological confirmation or laboratory-based follow-up using molecular diagnostic tools such as immune trypanolysis [32] which are extremely specific. The new model, therefore, fits specificity to the data using the literature estimates as a prior. Our initial assumption is that any false positives would be assigned in the staged data as stage 1.

Using the new model code and fitting procedure, our second step is to fit the model again to 2000–2013, making projections for 2014–2019 using the same assumptions about improvements to VC and PS in 2014 and 2015 respectively, specifically a 99% tsetse reduction after four months and a doubling of both the stage 1 and stage 2 passive detection rates. By comparing step 1 and 2 we can see the impact of model improvements, but not improvements due to more data availability. As with previous fitting, we fitted the same eight different model variants.

#### Step 3: New model fit for 2000–2019

Finally our third step is to fit the full data set including 2014–2019. This includes inferring (i) the stage 1 and stage 2 PS improvement from 2015 and (ii) the overall reduction of tsetse in the whole epidemiological focus (rather than using the measured tsetse density reduction in the Mandoul study area). Estimating the tsetse reduction using the model fitting, rather than substituting the value measured in the field allows us to test whether there is agreement between model outputs based on human case data and entomological dynamics observed in the study region. Any differences in these two could indicate that infection is occurring outside of the area under control by Tiny Targets. This third step results in the inference of three extra parameters, parameters $\eta _{H_{\mathrm{amp}}}$ and $\gamma _{H_{\mathrm{amp}}}$ which dictate the increased amplitude of the passive detection rate in stage 1 and stage 2 respectively, and the probability of a tsetse receiving a lethal dose of insecticide during the host-seeking phase of its feeding cycle, $p_{\text {targetdie}}$, for VC. See Additional file 1, section 2 for more model details.

Based on preliminary results which assumed false positives must be stage 1, we relaxed this assumption and allowed false positives to be reported as either stage 1 or stage 2 with a fitted probability.

As in the original study, CFSs were run for 2014–2019 to estimate the impact of intensified interventions on transmission and reporting. Since the actual strategy for this time period is now fitted rather than projected, this should provide a more robust estimate than reported previously.

Table 1 summarises assumptions for the three model fits described.

**Table 1:** Comparison of the fits of previous and new models

Changed items	Previous model	New model fit for 2000–2013	New model fit for 2000-2019
Active screening specificity	100%	Estimated from data	Estimated from data
False positives in active screening	No FP	FP in stage 1 only	FP in either stage
Passive detection improvement rate	Doubling of stage 1 and stage 2 PS detection rates from 2015	Doubling of stage 1 and stage 2 PS detection rates from 2015	Stage 1 and stage 2 PS detection rates from 2015 were estimated from the data
Underreporting	Both stages	Stage 2 only	Stage 2 only
Vector control reduction after 4 months	99%	99%	Total reduction estimated from the data

We describe the key differences between the previous Warwick gHAT model of Mahamat et al. [12] and the present study—the “new model”—when considering fits to previously available data (2000–2013) or the extended data sets (2000–2019)

*FP* false positive, *PS* passive screening

### Projections and cessation

Using the 2000–2019 model fit we also make projections until 2050 under five strategies (see Table 2), comprising of continued AS at either mean (during 2015–2019) or maximum coverage (during 2000–2019, MaxAS) with imperfect (< 100%, fitted) and perfect (= 100%) test specificity in conjunction with or without continuation of VC from 2021. In all scenarios PS was assumed to remain at present levels. Two MaxAS + VC strategies are presented in Additional file 1, section 5. Due to resource limitations, including costs and effort to implement such programmes, we also examined a cessation criterion for AS and VC following three years of zero case detections to consider the impact that stopping activities based on this measure could have (see Additional file 1 for sensitivity analysis on cessation criteria). The earliest cessation year was assumed to be 2021. Reactive AS takes place when any passive detections occur after the cessation criterion is met. The coverage of AS depends on its projection strategy. The duration of reactive AS depends on case reporting, such that reactive AS stops again when no case (either active or passive) is found in that year.

**Table 2:** Future strategies (2020 onwards) considered in the present study

Strategy name	AS coverage	PS	VC	Algorithm specificity (%)
MeanAS + VC (Imperfect Spec)	Mean of 2015–2019	Continued	Continued	$\approx$ 99.93
MeanAS + VC	Mean of 2015–2019	Continued	Continued	100
MeanAS	Mean of 2015–2019	Continued	Stopped in 2021	100
MaxAS	Max of 2000–2019	Continued	Stopped in 2021	100
Stop2021	None from 2021 (mean in 2020)	Continued	Stopped in 2021	100

This table shows the five possible future strategies we simulated using the ensemble model. We denote the coverage of AS, assumptions around PS detection rates, the use of VC and the specificity of the AS algorithm in defining cases in the different columns

*AS* active screening, *PS* passive screening, *VC* vector control, *Max* maximum

## Results

### Assessment of previous and new model fits to data

The previous model predicted that there would be a median of zero cases by 2017 in AS and by 2018 in PS. Newly available data from 2017 to 2019 are very low, but cases were still reported, indicating that our previous model overestimated the impact of the strategy on case reporting from 2014. Correcting for the real screening coverage had little impact on our predicted cases for this time period, due to the very limited remaining infection estimated by the model.

**Fig. 1:**
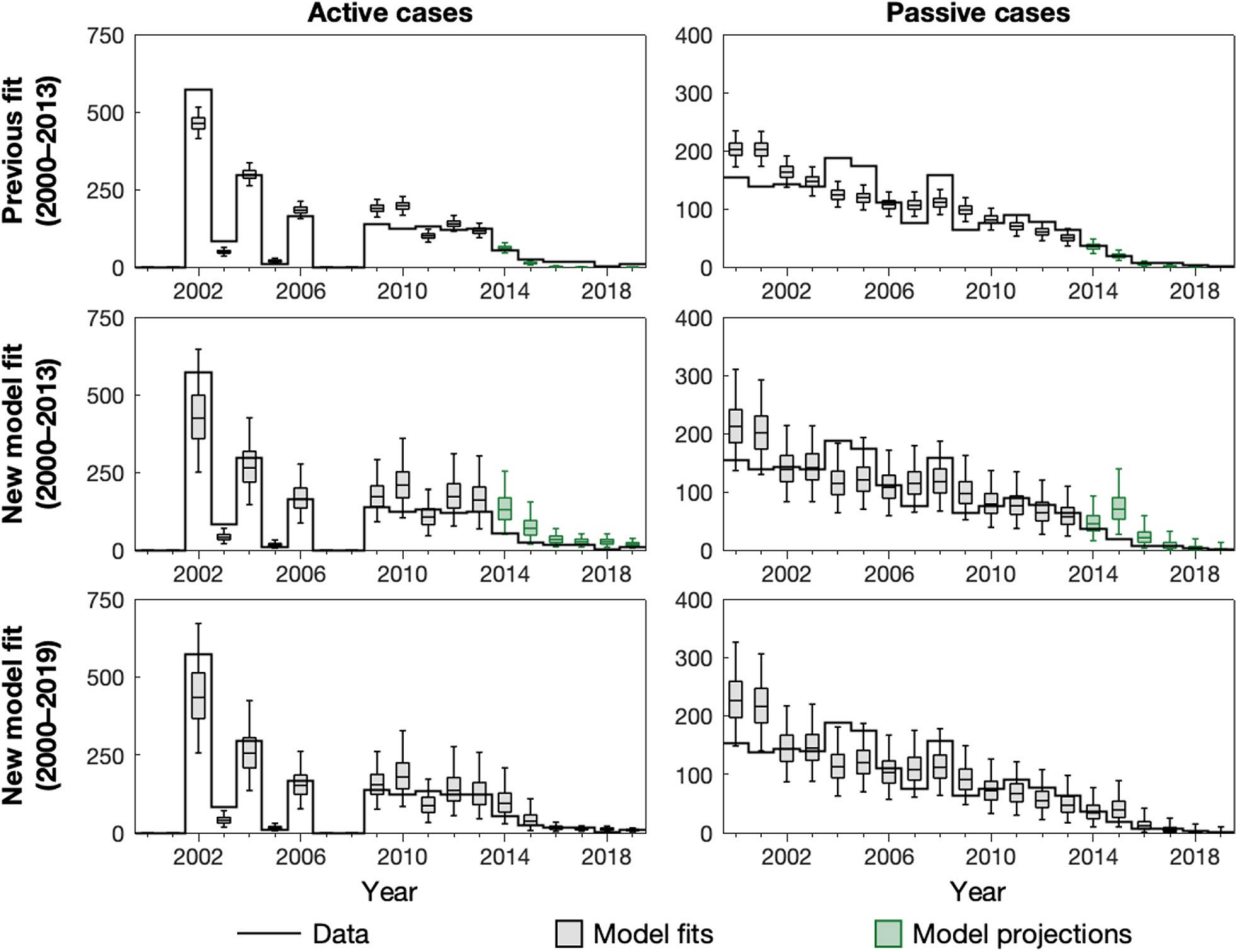
Comparison of previous and new model outputs. This figure panel shows the results of fitting to case data during **A** 2000–2013 using the previous model, **B** 2000–2013 using the new model, and **C** 2000–2019 using the new model. The solid black lines show the case data. Grey box and whiskers indicate years of model fits (median for centre line and 95% credible intervals for whiskers) and green box and whiskers denote model projections based on known active screening coverage

The updated model fit for the same fitted time period as before (2000–2013) better captures variation in case observations during the training period, attributed to the inclusion of overdispersion parameters in the updated model and improvement in the automated Markov chain Monte Marlo (MCMC) algorithm used for model fitting. It also matches more closely with the 2014–2019 data despite having no more information than the previous model for fitting and making the same assumptions on the VC reduction and passive detection rate improvement. Again, the overdispersion in the updated model leads to wider prediction intervals, more reflective of our uncertainty. In addition, the model now accounts for imperfect specificity in the diagnostic algorithm; not only does that allow for the potential of some false positives in 2017–2019, but it also impacts the model fit for 2000–2013. Median active detection predictions increased to 26, 26 and 18 for 2017–2019 compared to zero for the previous model with new screening data, and median passive detection predictions increased to 7, 2 and 0 from 1, 0 and 0 for the same time frame. The updated fit is therefore more in line with real data from these years (actively reported cases were 15, 3 and 10, and passively reported cases were 7, 3 and 1 for 2017–2019). Furthermore the enlarged prediction intervals for case reporting in the updated model now cover all the reported data for the prediction period except passive cases in 2015 and active cases in 2018.

The number of new infections per year (a measure of transmission) is a quantity which is very important with respect to the 2030 elimination goal, but ultimately is very difficult to measure directly in the field. The mechanistic model types used in this study and Mahamat et al. [12] can provide an estimate of transmission levels that would be required in order to generate the observed case reporting in active and passive detection. Factors such as underreporting and accuracy of diagnostic screening tests can impact the inferred level of transmission. Model estimates for transmission can be seen Additional file 1: Figs. S5–S7, and demonstrate that whilst the updated model fitted to the full data set infers slightly lower transmission in the early 2000s, the decrease between 2000 and 2013 follows the same trend (72% median reduction in the previous model and 68% median reduction in the updated model fitted to the 2000–2019 data). Imperfect specificity of the diagnostic algorithm captured in the updated model (but not the original) is one explanation for lower new infections; if some reported cases were false positive then the infections would be expected to be less than if all case reporting were true positives. The reporting parameter, *u*, can also impact inference of new infections. In the updated model fit (2000–2013), *u* was found to be around 20–33%, slightly higher than previously estimated using the same data (95% *CI* 16.8–23.6%, *u* = 19.1%). Analogous to imperfect specificity, lower underreporting of passive cases means that for the same number of new infections there would be more passive cases, or conversely, for the same number of passive cases there are actually fewer infections.

Despite the good agreement between aggregated active cases in the data and model (2000–2013) fit for the projection period, the model predicted that almost all reported cases would be false-positive from 2015 onward, whereas there are similar case detections in stage 1 as in stage 2 in the data. Therefore in the full data set fit (2000–2019) we decided to allow for false positive detections to be assigned to either stage, rather than only stage 1. Using the data from 2000–2013, the reported cases were too high to be able to estimate this trend as most would be expected to be true positive cases.

The updated model fitted to the full data set (2000–2019) enabled us to estimate the reduction in vectors in 2014 and the improvement to passive detection rates from 2015. Through our model calibration we found that the focus-wide reduction of tsetse (after 4 months) was estimated to be 99.1% (95% *CI* 96.1–99.6%), around our 99% assumed estimate based on the catch of tsetse from monitoring traps in the intervention area. For passive detection we estimated that stage 1 rate ($\eta _H$) increased to 2.12 times previous levels (95% *CI* 1.19–4.06) and stage 2 rate ($\gamma _H$) was 1.52 times previous levels (95% *CI* 1.04–8.60). This suggests that the doubling assumed for stage 1 passive detection in the previous model was quite reasonable, although it was slightly high for stage 2.

Using this full fit we estimated that the AS algorithm had a specificity of 99.9% (95% *CI* 99.9–100%) and that false positive cases were slightly more likely to be diagnosed as stage 2 rather than stage 1 (95% *CI* 0.46–0.74, Proportion = 0.61). Using the information directly from the data collected during 2016–2019 we can see that, proportionally, many of the cases reported in AS had positive serology but were not confirmed through parasitology (see Additional file 1: Fig. S10). Full posterior distributions for these and other model parameters can be found in Additional file 1: Table S8 and Fig. S3. One other estimated parameter was the basic reproduction number, $R_0$, which is a metric of potential for infection to spread. Here was estimated $R_0$ in the Mandoul focus to be 1.06 (95% *CI* 1.04–1.10) in the absence of all interventions other than basic PS. This value is comparable to estimates for other gHAT foci that have been quantified—with a value only slightly exceeding the critical threshold of one, that is required to sustain endemic levels of infection [25, 28, 33].

### Estimating past impact using counterfactual scenarios

By simulating CFSs without one or both intensified interventions occurring in 2014 and 2015 (VC and improved PS respectively), the model can be used to estimate case reporting and transmission that would have likely occurred if previous AS and baseline PS had continued. Table 3 shows the reduction in transmission for the first 2-year period and first 6-year period following implementation of intensified interventions. It also provides the percentage attributions of the basic strategy, enhanced PS and VC in achieving the reductions. Notably, VC is computed to have had the most impact on transmission amongst all the interventions for both time periods, however both intensified interventions were inferred to have substantial impact over the 6-year period (23.6% for enhanced PS and 34.7% for VC). Even without new interventions, AS and PS alone would have likely reduced transmission, but not enough to interrupt transmission by the target year of 2030 (see Additional file 1: Fig. S8); under the strategy including enhanced PS but no VC, there was a prediction of a 6.6% probability of EOT when performed in conjunction with mean AS. With the conducted intervention the estimated year of EOT was 2015 (95% *CI* 2015–2015).

**Table 3:** Estimated percentage reduction in transmission by intervention since intensified strategy began

Transmission reduction by intervention	Transmission	Transmission
	2013–2015	2013–2019
Total reduction (%)	100.0 (99.7–100.0)	100.0 (100.0–100.0)
Percentage of reduction attributed to AS and baseline PS	19.9 (12.8–28.5)	41.3 (27.4–55.7)
Percentage of reduction attributed to enhanced PS	5.6 (1.2–18.3)	23.6 (8.3–43.8)
Percentage of reduction attributed to VC	74.0 (57.8–84.4)	34.7 (13.9–58.2)

Attributions to each strategy component are based on counterfactual strategy simulations. Medians are given with 95% credible intervals in brackets

*AS* active screening, *PS* passive screening, *VC* vector control

### Evidence for alternative model structures (including animal reservoirs)

We found that there was most support for model variants 4 and 5—both of which have high-/low-risk structure for the human population, but do not have animal reservoirs (see Additional file 1 for more details). Less than 0.1% of the ensemble model was made up of simulations from Models 7 and 8 (equivalent to Models 4 and 5 but including transmission to and from animals). Even taking these models alone, fitting indicates it is unlikely that animals constitute a maintenance reservoir, with 39% and 24% of simulations having human-only maintenance of infection in Models 7 and 8 respectively, and the remainder requiring both transmission from humans and non-human animals.

### Model projections to 2030

**Fig. 2:**
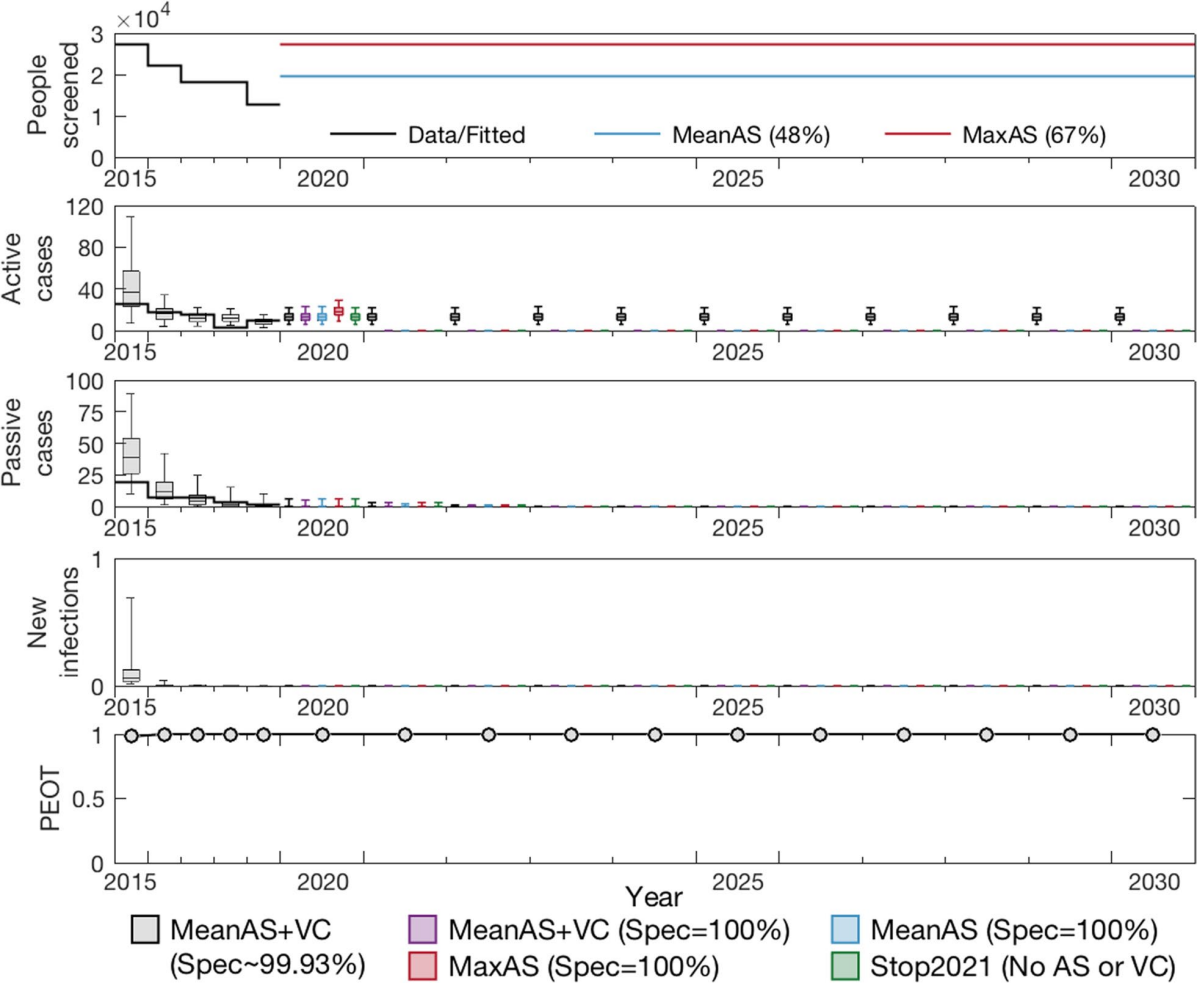
Projections to 2030. The ensemble model fitted to data during 2000–2019 was used to make projections under five different strategies. The baseline strategy, MeanAS + VC with imperfect specificity ($\sim 99.93\%$), is denoted by grey boxes. With specificity improved to 100% from 2021, the strategy MeanAS + VC is denoted by purple boxes. Blue and red boxes are MeanAS and MaxAS strategies with AS screening specificity switching to 100% and stopping VC from 2021. Finally, Stop 2021 under which both AS and VC stop in 2021 is shown by the green boxes. All simulations assume PS remains at the level as estimated for 2019 and continues indefinitely. The top panel shows the level of AS assumed in the different projections, the second row shows the active case predictions, the third shows the passive case predictions and the forth shows the expected amount of new infections. The bottom row shows the probability of EOT for each year. *AS*: active screening; *VC*: vector control; *Max*: maximum; *EOT*: elimination of transmission

Figure 2 shows the ensemble model prediction (using the updated 2000–2019 fit) under five different strategies including three with continuation of the mean level of AS (2015–2019), one under the maximum coverage observed during 2000–2019, and one with no AS from 2021. In the baseline case we assume that specificity remains imperfect in AS (99.9%), however in all other scenarios we assume that additional testing in the AS algorithm increases specificity to 100% from 2021. We simulate cessation of AS following three years of no case detections in Fig. 2, however other cessation criteria yield virtually identical results.

Notably, under all strategies we predict that EOT has already occurred and will not resume even after cessation of vertical interventions. In these simulations the extreme suppression of the fly population does not recover, however even if there were very rapid bounce back of tsetse through reinvasion or other means, only 2% of our model simulations (with 90% adult tsetse reintroduction in 2021) saw resurgence of infection in humans where transmission occurred beyond 2030 as a result. Our more modest reintroduction scenarios (10% and 50% in 2021) found 0% and 1% resurgence probability respectively (see Additional file 1: Fig. S9).

Interactive results for both the CFSs and the projections from 2020 can be found through our graphical user interface (https://hatmepp.warwick.ac.uk/Mandoulfitandproject/v1/), which shows the ensemble model results for both sets of model outputs.

## Discussion

This modelling study has critically examined previous modelling work to assess its predictive ability and to refine the results presented before [12]. Overall, we have found that the model predictions were reasonable, however slightly underestimated active case reporting in the late 2010s. A combination of model improvements and new data have allowed us to examine how predictions could be updated using refined methodology alone, and what additional information could be learnt from new data. Our updated model estimates the same EOT year as before (2015) and the previous and updated models attribute transmission reductions in quantitatively similar proportions to the different intervention activities. One particular challenge faced in the previous modelling exercise was uncertainty in the future level of AS coverage that would occur between 2016–2019. In hindsight the level used (27,265, which corresponded to the coverage in 2015) for the Mahamat et al. projections was higher than achieved, and screening dropped in subsequent years. Despite this, we don’t think that in this instance the screening level substantially impacted our results, as is demonstrated with our re-simulation of the original gHAT model with correct screening coverage for those years (see Fig. 1). Whilst the new modelling presented here now utilises the 2016–2019 screening data for both fitting and subsequent projections, predictive modelling always faces the challenge of defining realistic future intervention strategies as there are scenarios where changing to a different strategy than the one modelled can have a major impact on results. This type of effect has been particularly noticeable in model projections or forecasting for the COVID-19 pandemic and policy rapidly evolves and is often not the same as interventions that we used to create model projections [34]. Here we try to mitigate against this situation by providing projections for five alternative strategies which we believe provide a good insight into a range of plausible outcomes.

This modelling update brings together quantitative validation with discussions for those familiar with on-the-ground implementation, thus representing an important step for models aimed at providing policy recommendations. The present study adheres to the policy-relevant items for reporting models in epidemiology of neglected tropical diseases (NTD-PRIME) criteria, which map good modelling practice [24] (see Additional file 1 for more details). Our graphical user interface also provides a more interactive way to view our new results https://hatmepp.warwick.ac.uk/Mandoulfitandproject/v1/.

### Limitations

Despite our updated model modification to better reflect observed cases (e.g. overdispersion in sampling and the possibility of false positive reporting), there are still elements of the biology which are not captured, including no possibility for asymptomatic, self-curing human infections [35] and no imported cases from other regions. We use a deterministic model which captures the average dynamics rather than a stochastic model, although work using both deterministic and stochastic gHAT models in other regions [36–39] indicates that we would expect the dynamics to be very similar. Future work regarding stochastic reinvasion of infection via human imports (such as from other extant foci in Chad or over the nearby border with the Central African Republic) would provide insights on post-elimination risks for Mandoul, but is beyond the scope of the present study.

This study examined a variety of plausible future intervention strategies for Mandoul, however PS was assumed to remain intact at current operating levels in all of them. Reduction in the capacity or coverage to find and treat gHAT-infected people could have deleterious impact on the focus which otherwise anticipates reaching zero infected people in the next few years. Another concern is the possible effect of other infectious disease outbreaks on gHAT. The most notable example is in Guinea, where the West African Ebola outbreak in 2014–2016 had a large impact on the national control programme’s active and PS activities and is likely to have increased morbidity and mortality from this disease [18, 40]. Whilst there was some delay to AS activities for gHAT in Chad in 2020 due to the COVID-19 pandemic, screening was able to resume later in the year yielding only a small impact on total annual coverage compared to recent years. Other factors which could lead to reduction in PS capacity include lack of funding, motivation, or dedicated human resources especially after several years of zero case detections. Horizontal integration within the health system could be vital for post-elimination monitoring and signs of resurgence.

Ongoing work is bringing together transmission modelling and costs in a health economic framework in order to provide assessment of the expected total costs and cost-effectiveness of possible future strategies in the focus.

## Conclusions

In this study we used a mathematical transmission model of gHAT in Mandoul to update previous modelling including demonstrating the impact of improvements made, and using new data to further improve the updated model fit. We found that our previous assumption of a doubling stage 1 passive detection rate appears appropriate, although the stage 2 passive detection rate is unlikely to have increased substantially. Tsetse reduction estimated by the model was in good agreement with that measured by tsetse density in the intervention area and tsetse control contributed to 74.0% of the transmission reduction between 2013–2015. Modelling indicated that elimination of transmission occurred in 2015.

Projections suggest that we expect to have zero passive case reporting before 2023, although active case detection would continue due to imperfect specificity of the current diagnostic algorithm, and therefore additional confirmatory testing (such as mAECT, LAMP or trypanolysis) ought to be considered. It appears that cessation of vertical interventions (active screening and vector control) would be unlikely to result in a resurgence of infection if there are no or few importations of gHAT to the region, however continued surveillance will be important to have assurance that elimination of transmission has been met.

## Supplementary information

**Additional file 1.** Additional model description, results and PRIME-NTD criteria checklist.

## Data Availability

Data cannot be shared publicly because they were aggregated from the World Health Organisation’s HAT Atlas which is under the stewardship of the WHO. Data are available from the WHO (contact neglected.diseases@who.int or visit https://www.who.int/trypanosomiasis_african/country/foci_AFRO/en/) for researchers who meet the criteria for access. Model code and outputs produced from this study are available through Open Science Framework https://osf.io/rak9d/.

## Abbreviations

AS: Active screening
CATT: Card agglutination test for trypanosomiasis
COVID-19: Coronavirus disease 2019
CFSs: Counterfactual scenarios
*CI*: Credible interval
CSF: Cerebrospinal fluid
DRC: Democratic Republic of Congo
EOT: Elimination of transmission
FP: False positive
gHAT: *gambiense* human African trypanosomiasis
LAMP: Loop-mediated isothermal amplification
mAECT: Mini anion exchange centrifugation technique
MCMC: Markov chain Monte Carlo
NECT: Nifurtimox–eflornithine combination therapy
NTDs: Neglected tropical diseases
PNLTHA-Chad: Programme National de lutte contre la Trypanosomiase Humaine Africaine (National control programme against human African trypanosomiasis )
PS: Passive screening
RDT: Rapid diagnostic test
VC: Vector control
WHO: World Health Organization
